# Integrative metabolomics and proteomics reveal the effects and mechanisms of *Salvia miltiorrhiza* in alleviating traumatic blood stasis syndrome

**DOI:** 10.3389/fvets.2025.1579790

**Published:** 2025-04-25

**Authors:** Pei Guo, Xin Wang, Qi Chen, Xufeng Dong, Zhihua Qin, Jiaguo Liu

**Affiliations:** ^1^College of Veterinary Medicine, Nanjing Agricultural University, Nanjing, China; ^2^College of Veterinary Medicine, Qingdao Agricultural University, Qingdao, China; ^3^Gaomi Animal Husbandry Development Center, Shandong, China

**Keywords:** *Salvia miltiorrhiza*, traumatic blood stasis syndrome, platelet, proteomics, metabolomics

## Abstract

*Salvia miltiorrhiza* (SM) is widely used in clinical practice for the treatment of cardiovascular diseases. However, the efficacy and mechanisms of SM in addressing traumatic blood stasis syndrome (TBSS) have not been thoroughly investigated. We established a TBSS model in cats and examined the muscle swelling rate (MSR), pain index, coagulation index, hematological parameters, inflammatory factors, and platelet function levels to assess the effects of SM. Subsequently, integrative metabolomics and proteomics were employed to elucidate the effects and mechanisms of SM in alleviating TBSS. The results demonstrate that the effect of SM was evaluated by establishing a cat model of TBSS. Administration of SM for 10 days significant decrease in markers such as MSR, pain index, WBC, PLT, PCT, FIB, PAI-1, TNF-*α*, IL-6, IL-1*β*, TXB_2_, TXB_2_/6-Keto-PGF1α, β-TG, and PF4. Additionally, there was a significant increase in APTT, PT, TT, t-PA, IL-10, 6-Keto-PGF1α, and FN. These findings suggest that SM regulates swelling and pain, inflammatory responses, coagulation and fibrinolytic system abnormalities, as well as platelet aggregation and activation. Through platelet metabolomic and proteomic analyses, it was found that SM inhibited the aggregation and activation processes of TBSS platelets by modulating physiological pathways, including tryptophan metabolism, purine metabolism, fatty acid metabolism, the complement and coagulation cascades, and platelet activation.

## 1 Introduction

As people increasingly prioritize the healthy lifestyles of their pets, the frequency of outdoor activities and exercise for these animals has risen. However, this trend also elevates the risk of injuries, which may result from collisions, falls, or improper exercise. TBSS is a common sports injury frequently encountered in clinical practice, significantly impacting both physical and mental health in pets and their owners (1). TBSS can manifest in various ways, including localized edema, muscle fiber rupture, pain, bruising, ecchymosis, and dysfunction. Following a soft tissue injury, the body initiates a repair process through an inflammatory response. According to TCM, syndrome differentiation of TBSS indicates that qi stagnation and blood stasis occur in the early stages of injury. Symptoms may include localized swelling, tingling, and fixed pain, often accompanied by visible blue and purple blood spots or large hematomas. In the later stages of injury, blood deficiency and cold coagulation types may manifest, primarily characterized by pain, swelling, and mild tenderness. The treatment principle focuses on promoting blood circulation, removing blood stasis, reducing swelling, and alleviating pain (2).

The fundamental basis of blood stasis syndrome (BSS) lies in the damage to vascular endothelial cells, with platelet activation playing a pivotal role in this process. This activation significantly influences the progression of endothelial cell injury and the eventual manifestation of BSS. Initially, research on the pathological mechanisms underlying blood stasis syndrome primarily focused on the functions of platelets and endothelial cells. However, as investigations have progressed, it has become increasingly evident that the development of this syndrome is a highly intricate and gradual biological process, meticulously regulated by numerous factors and pathways. At the molecular level, this process involves alterations in gene expression, protein synthesis and modification, as well as dynamic shifts in metabolic profiles. The rapid advancement of modern omics methodologies has led to the emergence of metabolomics and proteomics as crucial tools for elucidating the mechanisms of TCM. These methodologies provide a more comprehensive understanding of the pharmacological impacts of bioactive constituents (3, 4). Metabolomics enables in-depth analysis of metabolites and identification of bioactive compounds (5), while proteomics complements metabolomic findings by clarifying changes in interactions and pathways, thereby validating pharmacological effects (6).

SM refers to the dried root and root stem of *Salvia miltiorrhiza* Bge. It has a bitter taste and is considered slightly cold in nature, affecting the heart and liver meridians. SM is widely used in the treatment of cardiovascular and cerebrovascular diseases, addressing issues such as vascular endothelial cell injury (7), abnormal coagulation and fibrinolysis (8), inhibition of platelet activation and aggregation (9), vascular dilation (10), and dyslipidemia (11), among others (12). SM has been reported in the literature for the treatment of blood stasis syndrome (12). Our previous studies have detailed the preparation method for SM chewable tablets and established quality standards (13). However, the efficacy and mechanisms of SM in treating TBSS have not been thoroughly investigated. We integrated proteomics and metabolomics to elucidate the regulatory mechanisms by which SM alleviates TBSS.

## 2 Materials and methods

### 2.1 Drugs and reagents

SM tablet was formulated from the TCM *Salvia miltiorrhiza*, which was sourced from the Anguo Traditional Chinese Medicine Market in Hebei, China. The production process of the SM tablet involved extraction, concentration, drying, granulation, and tablet pressing. The final product contains 14.35 mg/g of salvianolic acid B (Sal B).

Sanqi Tablet (SQ) is a novel veterinary drug that was approved in 2021 by the Ministry of Agriculture and Rural Affairs of the People’s Republic of China. It is manufactured by Beijing Center Technology Co., Ltd. This medication has multiple functions, including promoting blood circulation, stopping bleeding, reducing swelling, and alleviating pain. It is specifically designed to relieve swelling and pain associated with soft tissue injuries in pets.

A cat ELISA kit for detecting cytokines (IL-1*β*, IL-10, TNF-*α*, IL-6), as well as assay kits for 6-keto-PGF1α, TXB_2_, FN, β-TG, PF4, PAI-1, and t-PA was purchased from Nanjing Jiancheng Bioengineering Institute.

### 2.2 Animal experiments and study design

Twelve healthy adult male cats, aged 1 to 3 years and weighing between 2.45 and 3.65 kg, were housed together in a single cage within the experimental animal facility. The facility maintained a temperature range of 20 to 25°C and a relative humidity of 40 to 60%. The cats were provided with access to clean drinking water and cat food that complied with the guidelines established by the Ethics Committee for Laboratory Animals.

An enhanced method was utilized, which involved padding and securing the right hind limb of each cat with gauze to prevent fractures. A 200 g weight was dropped freely from a height of 50 cm to strike the midsection of the right hind limb muscle at a consistent location. Following the modeling procedure, there was no evidence of local soft tissue rupture, no bone fractures in the limb, and swelling was observed in the middle of the muscle, indicating successful TBSS modeling.

Nine healthy and successful TBSS modeling cats were randomly divided into three groups: TBSS, SM, and SQ. The SQ group received daily oral administration of 0.12 g/kg of body weight, while the SM group received 0.42 g/kg, with both treatments administered for a duration of 10 days. EDTA anticoagulated blood samples were collected from the forelimbs on 0, 5, and 10 days. A portion of the samples was analyzed using an automatic blood analyzer to assess the routine blood indices of each group. The remaining plasma was obtained by centrifugation at 3500 rpm for 15 min. The levels of t-PA, PAI-1, TAI, TNF-*α*, IL-6, IL-1*β*, IL-10, 6-keto-PGF1α, TXB_2_, FN, β-TG, and TF4 were measured using an ELISA test kit.

### 2.3 Metabolomics analysis

#### 2.3.1 Platelet collection and preparation

EDTA were collected from the forelimb vein and centrifuged at 1500 rpm for 15 min. This process resulted in the separation of the upper layer containing platelet-rich plasma, while white blood cells and red blood cells settled in the lower layer. The platelet-rich plasma was then transferred to a centrifuge tube and subjected to a second centrifugation at 3500 rpm for 8 min, causing the platelets to precipitate at the bottom. The precipitated platelets were subsequently frozen in liquid nitrogen.

Platelet samples from the NC, TBSS, and SM groups (*n* = 3) were thawed at room temperature and thoroughly mixed for metabolite extraction. The extract was combined with four volumes of a methanol-acetonitrile mixture (1:1, v/v), mixed for 30 s, sonicated in an ice water bath for 10 min, and then incubated at −40°C for 1 h to precipitate proteins. The sample was centrifuged at 4°C, 12,000 rpm (RCF = 13,800 g, R = 8.6 cm) for 15 min, and the supernatant was transferred to an injection vial for analysis.

#### 2.3.2 LC–MS conditions

Using a Vanquish UPLC equipped with a BEH C18 column (2.1 mm × 50 mm, 1.7 μm, Waters, United States), the mobile phase consisted of 25 mM ammonium acetate and 25 mM ammonium hydroxide in water (pH 9.75; A) and acetonitrile (B). The elution gradient was delivered at a flow rate of 0.5 mL/min and was as follows: at 0 min, 95% B; at 7 min, 65% B; at 9 min, 40% B; at 9.1 min, 95% B; and at 12 min, 95% B. The injection volume was 2 μL, and the sample disk temperature was maintained at 4°C.

First-order and second-order mass spectrometry data acquisition was conducted using an Orbitrap Exploris 120 mass spectrometer. The parameters for the ion source (ESI) were set as follows: sheath gas flow rate: 50 Arb; auxiliary air velocity: 15 Arb; transmission tube temperature: 320°C; full MS resolution: 60,000; MS/MS resolution: 15,000; collision energy: SNCE 20/30/40; and spray voltage: 3.8 kV (positive) or −3.4 kV (negative).

#### 2.3.3 Metabolomics data analysis

The screening criteria for differential metabolites included a Variable Importance in Projection (VIP) score of 1 for the first principal component of the Orthogonal Partial Least Squares Discriminant Analysis (OPLS-DA) model, along with a *p*-value <0.05. KEGG pathway enrichment analysis was conducted using the MetaboAnalyst platform, where significantly divergent metabolites were annotated, classified, and subjected to pathway enrichment based on the KEGG database.

### 2.4 Proteomics analysis

#### 2.4.1 Protein extraction and enzymatic digestion

Samples were thawed on ice and mixed at 4°C at 1100 g for 20 min. 5 μL of the supernatant were taken and combined with 195 μL of 8 M UA buffer (8 M urea, 150 mM Tris–HCl, pH 8.0). The mixture was briefly centrifuged, and 10 μL of the extracted protein concentration was determined using the Bradford method. The remaining sample was frozen at −80°C.

Using a micro/universal proteome digestion kit, 20 μL of protein was added to the eight-row containing MMB beads and incubated at 37°C for 30 min. Following this, 45 μL of binding buffer was added and allowed to react for 15 min. After incubation, the supernatant was discarded, and the MMB beads were washed three times with washing buffer. Subsequently, 20 μL of resuspension buffer was added to the magnetic beads, and they were incubated at 37°C for more than 4 h. After this incubation, an additional 5 μL of quench buffer was added to halt the digestion process, and the mixture was lyophilized.

#### 2.4.2 High pH RP-UPLC separation

An equal amount of enzymatic samples was mixed. The samples were centrifuged at 14,000 g for 20 min, and the supernatant was collected. Mobile phase A, consisting of 100% water and 0.1% formic acid, was prepared for use in a reverse-phase C18 column. The elution was conducted with a flow rate gradient of 0.7 mL/min as follows: from 0 to 5 min, 5% mobile phase B (80% acetonitrile, 0.1% formic acid) was used for equilibration; from 5 to 40 min, the concentration of mobile phase B was increased to 8%; from 40 to 62 min, it was further increased to 18%; from 62 to 64 min, it reached 32%; from 64 to 72 min, it was raised to 95%; and finally, from 72 to 80 min, the system was equilibrated back to 5% mobile phase B for 8 min. The eluting peaks were monitored, fractions were collected, and the samples were subsequently frozen for storage.

#### 2.4.3 LC–MS/MS analysis

The 2.4.2 freeze-dried samples were reconstituted with mobile phase A, then centrifuged at 14,000 g for 20 min at 4°C. A total of 400 ng of the supernatant was injected. The PLC was eluted at a flow rate of 500 nL/min, with the following separation gradient: 0–17 min at 3.5% mobile phase B; 17–18 min at 32% mobile phase B; 18–21 min at 95% mobile phase B; and 21–22 min at 1% mobile phase B. Using the timsTOF_HT mass spectrometer equipped with a Captive Spray ion source, data were collected in DIA mode. The mass spectrometer was set to a scan range of m/z 300–1,500, with the primary MS resolution set at 60,000 (at 1222 m/z). In the TIMS tunnel, the accumulation time was set to 50 ms, the capillary voltage was set to 1.5 kV, and the mobility ranged from 0.70 to 1.30 cm^2^/(V). The total cycle time was 1.23 s to generate the MS detection raw data (.d).

#### 2.4.4 Proteomic data analysis

Multivariate statistical analysis, including principal component analysis (PCA) and hierarchical cluster analysis, was conducted using a T-test with the screening criteria of “FC > 1.2 or FC < 0.833 (1/1.2) and *p* < 0.05” to identify significant upregulation and downregulation of differential proteins. Additionally, KEGG and GO enrichment analyses were performed for the differential proteins.

### 2.5 Parallel reaction monitoring analysis

Based on the analysis of the target mass spectrum, peptide segments that exhibit reliable identification and optimal chromatographic separation for each target protein are selected for subsequent quantitative analysis. The quantitative data for the target protein is derived from the quantitative information of the selected peptide segments.

### 2.6 Statistical analysis

The data are presented as the mean±standard deviation (SD), and the analysis was conducted using GraphPad Prism version 8.0.2. The statistical analysis included analysis of variance (ANOVA) to compare groups, followed by Dunnett’s test. Statistical significance was defined as *p* < 0.05.

## 3 Results

### 3.1 SM treatment can alleviate MSR and blood routine abnormalities in TBSS cats

**Figure 1:**
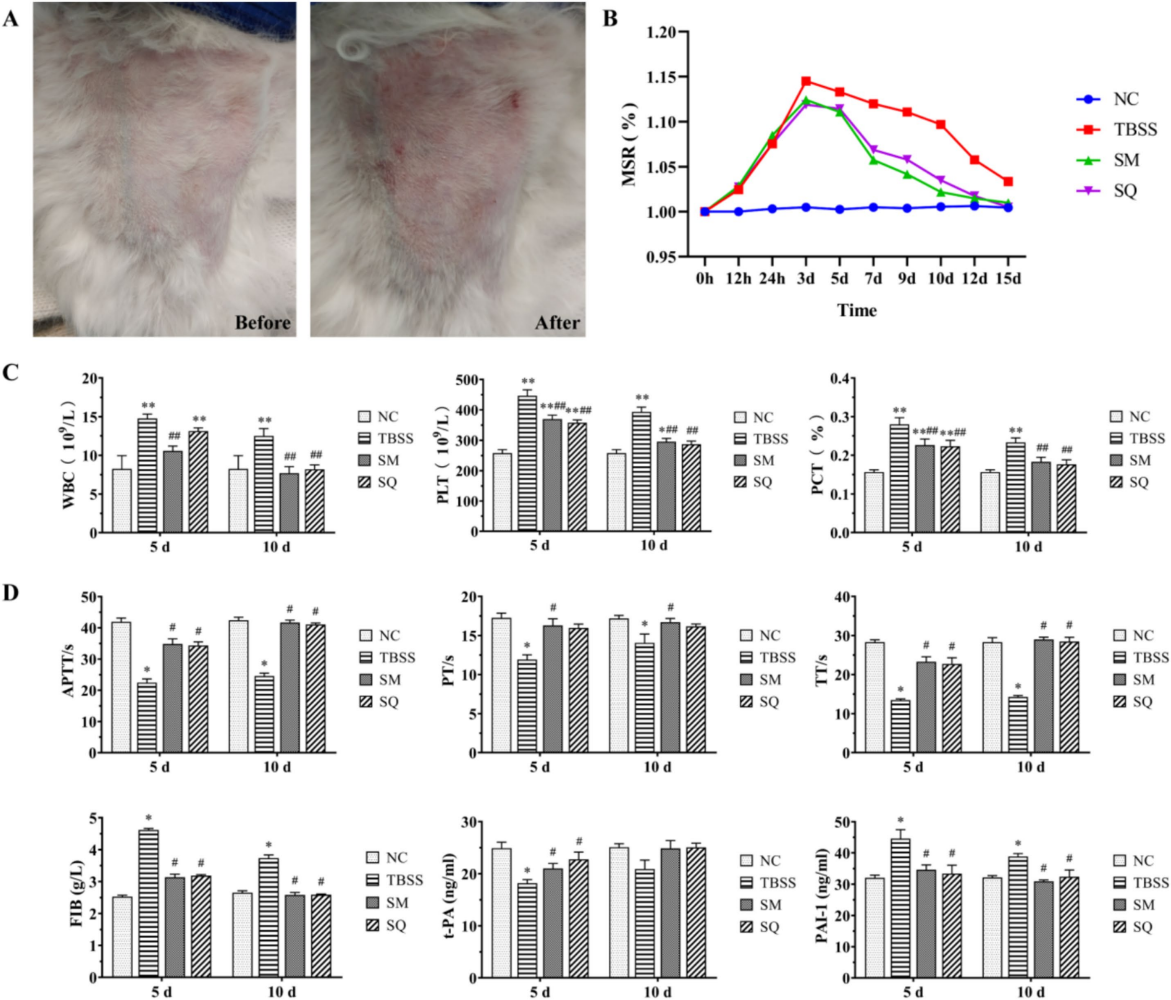
**(A)** Apparent changes in TBSS sites before and after mold exposure. **(B)** Results of MSR at various time points. **(C)** Effect of SM on four blood coagulation tests in cats. **(D)** Effect of SM on t-PA and PAI-1 levels in cats. Compared to the NC group, ^*^p < 0.05, ^**^p < 0.01. Compared to the TBSS group, ^#^*p* < 0.05, ^##^*p* < 0.01. The same applies to the subsequent comparisons.

In this experiment, the cat TBSS model was established by applying heavy objects. The observable changes in blood stasis sites before and after mold formation are illustrated in Figure 1A. Notable ecchymosis, petechiae, and swelling were evident following mold formation, indicating the success of the model in this experiment. During the test, TBSS cats exhibited reduced spontaneous activity and lower food and water intake compared to the normal control (NC) group.

The results of the MSR are presented in Figure 1B. The TBSS group exhibited an increase in MSR (*p* < 0.05) and reached peak swelling between 24 h and 3 days compared to the NC group. The MSR was significantly lower after both SM and SQ administration when compared to the TBSS group (*p* > 0.05). As shown in Figure 1C, at 5 and 10 days, WBC, PLT, and PCT levels were all significantly higher in the TBSS group compared to the NC group (*p* < 0.01), indicating that the traumatic blood stasis model had a significant impact on the routine blood indices of cats. Additionally, WBC, PLT, and PCT levels were significantly lower in the SM group compared to the TBSS group (*p* < 0.01).

### 3.2 SM treatment can alleviate abnormalities in the coagulation-fibrinolytic system in TBSS cats

The results of the fibrinolysis-coagulation index are presented in Figure 1D. Compared to the NC group, the TBSS group exhibited significantly reduced levels of APTT, PT, TT, and t-PA (*p* < 0.05), while FIB and PAI-1 levels were significantly increased (*p* < 0.05). These findings indicate that coagulation time and function were enhanced, resulting in increased blood viscosity in the TBSS group. In contrast, when compared to the TBSS group, APTT, PT, TT, and t-PA levels were significantly increased (*p* < 0.05), while FIB and PAI-1 levels were significantly decreased (*p* < 0.05). This suggests that SM administration improved the abnormal coagulation function induced by mold exposure and enhanced the activity of the fibrinolytic system, thereby alleviating blood stasis associated with TBSS.

### 3.3 SM treatment can alleviate inflammation in TBSS cats

**Figure 2:**
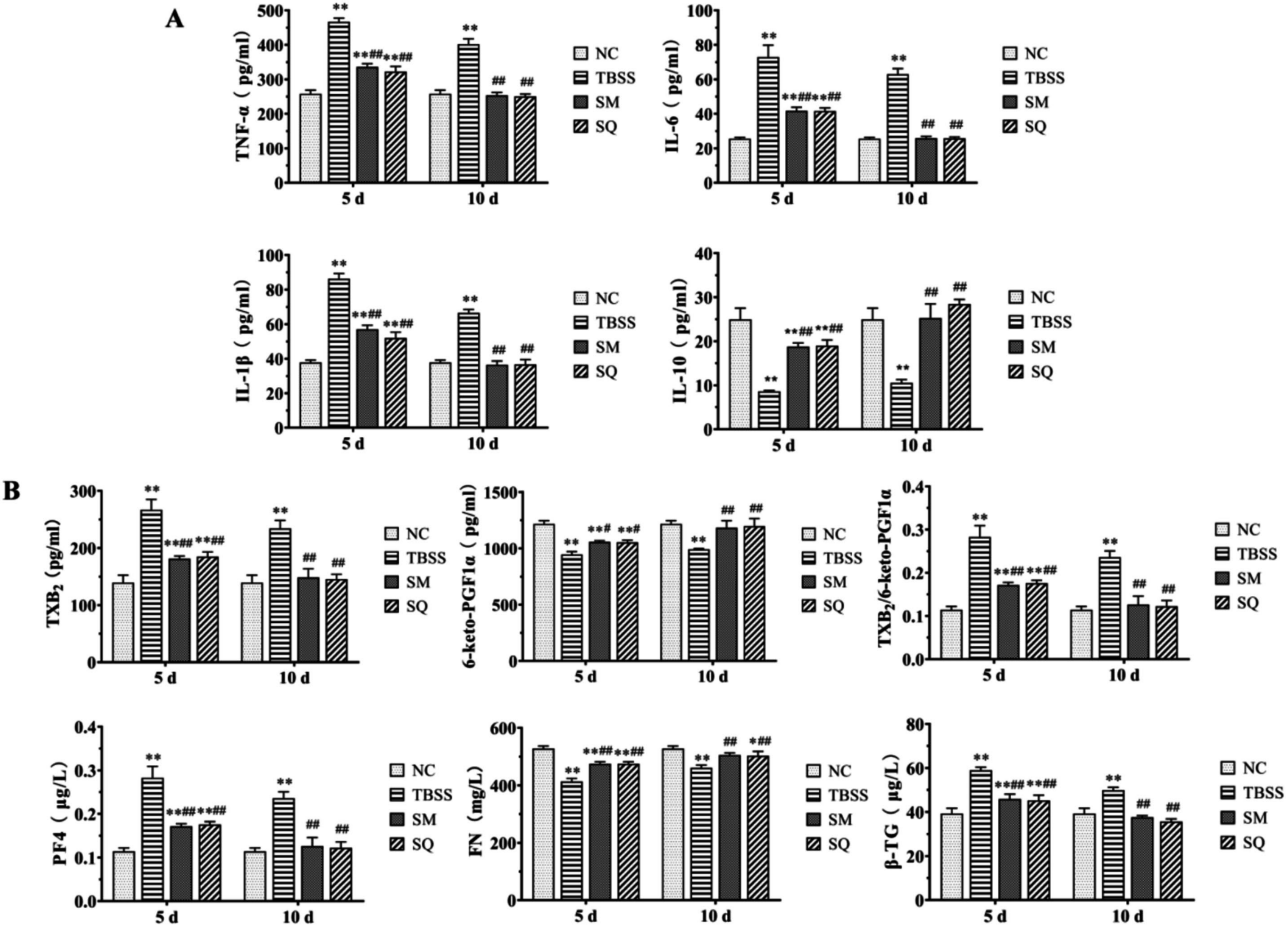
**(A)** Effect of SM on blood plasma levels of inflammatory factors in cats. **(B)** Effect of SM on blood plasma levels of platelet function factors in cats.

The results of the inflammatory factor level determination are presented in Figure 2A. Compared to the NC group, the levels of TNF-*α*, IL-1β, and IL-6 were significantly elevated in the TBSS group, while IL-10 showed a significant reduction (*p* < 0.01). This indicates that TBSS has a substantial impact on serum inflammatory factors in cats. After 5 and 10 days of SM and SQ administration, the levels of TNF-*α*, IL-1β, and IL-6 were significantly lower (*p* < 0.01) than those in the TBSS group, while IL-10 was significantly higher (*p* < 0.01). After 5 days of administration of SM and SQ, the levels of TNF-α, IL-1*β*, and IL-6 were significantly higher (*p* < 0.01) than those in the NC group, whereas the reduction in IL-10 was also significant (*p* < 0.01) compared to the NC group. Following 10 days of SM and SQ administration, the levels of TNF-α, IL-1β, IL-6, and IL-10 did not differ significantly from those in the NC group (*p* > 0.05). These findings suggest that SM administration mitigates the inflammatory response induced by TBSS.

### 3.4 SM treatment can alleviate abnormal platelet function in TBSS cats

The results of the cat platelet function indexes are shown in Figure 2B. Compared to the NC group, TXB_2_, *β*-TG, and PF4 were significantly elevated in the TBSS group (*p* < 0.01), while TXB_2_/6-Keto-PGF1α, as well as FN, and 6-keto-PGF1α were significantly reduced (*p* < 0.01). These findings indicate that TBSS has a substantial impact on cat platelet function. In comparison to the TBSS group, after 5 and 10 days, levels of TXB_2_, TXB_2_/6-Keto-PGF1α, *β*-TG, and PF4 were significantly lower (*p* < 0.01) than those in the TBSS group. Conversely, 6-Keto-PGF1α level was significantly higher (*p* < 0.05), and FN extremely significantly higher (*p* < 0.01) compared to the TBSS group. After 5 days of administration of SM and SQ, levels of TXB_2_, TXB_2_/6-Keto-PGF1α, β-TG, and PF4 were significantly higher (*p* < 0.01) than those in the NC group, while 6-Keto-PGF1α and FN levels were significantly lower (*p* < 0.01) compared to the NC group. After 10 days of SM and SQ administration, levels of TXB_2_, 6-Keto-PGF1α, TXB_2_/6-Keto-PGF1α, FN, β-TG, and PF4 did not differ significantly from those in the NC group (*p* > 0.05). This indicates that the platelet function abnormalities caused by mold formation were mitigated by SM administration.

### 3.5 Platelet metabolomic analysis

#### 3.5.1 Reliability assessment of the analytical method

Platelet Metabolomic Analysis: the TIC plot analysis reveals that the well-separated peaks are sharp and symmetrical (Supplementary Figure S1), indicating that the sample metabolites have met the conditions necessary for mass spectrometry quantification. Principal component analysis (PCA) was performed on the obtained data, with the results presented in Supplementary Figure S2. The PCA of the three samples in the NC, TBSS, and SM groups demonstrated that all samples fell within the 95% confidence interval. Differences in metabolite levels between groups were further assessed using orthogonal partial least squares discriminant analysis (OPLS-DA). As illustrated in Supplementary Figure S3, the variable importance in projection (VIP) was calculated through OPLS-DA to evaluate the influence of metabolites on the classification of each group, assist in identifying differential metabolites, and assess the significance of these metabolites between groups using a T-test. The screening criteria were set at VIP > 1 for the first principal component of the OPLS-DA model and a *p*-value < 0.05. Metabolites with a fold change (FC) > 1 were considered upregulated, while those with FC < 1 were classified as downregulated.

#### 3.5.2 Differential metabolite screening and enrichment analysis

**Figure 3:**
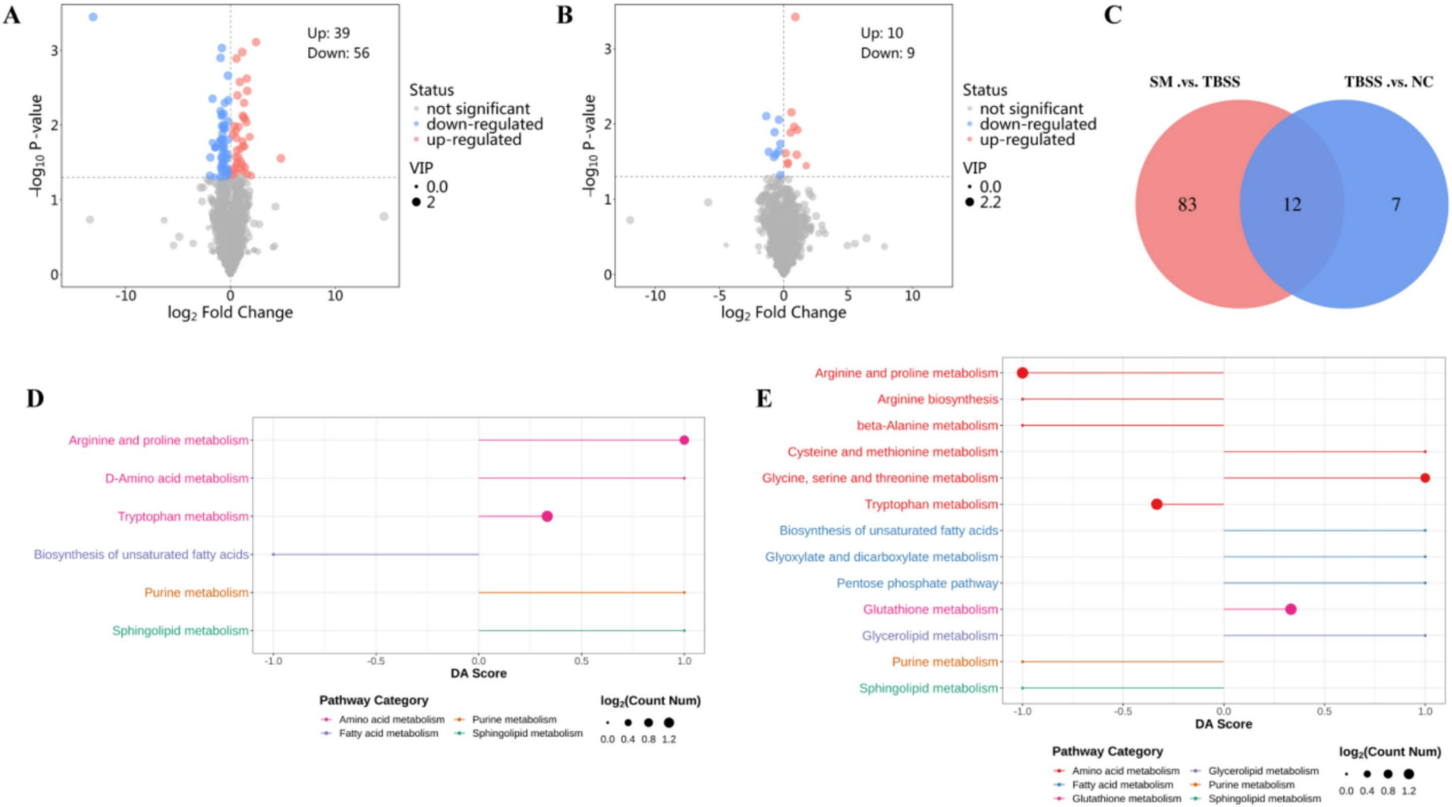
**(A,B)** Are volcano plots of TBSS vs. NC, SM vs. TBSS, respectively. Red indicates upregulated metabolites, while green indicates downregulated metabolites. **(C)** Displays the common differential metabolites between the two groups. **(D)** DA Score for the TBSS vs. NC group metabolites. **(E)** DA Score for the SM vs. TBSS group metabolites.

TBSS vs. NC identified 19 differential metabolites, with 10 being upregulated and 9 downregulated (Figure 3A). In contrast, SM vs. TBSS revealed 95 differentially expressed proteins, of which 39 were upregulated and 56 were downregulated (Figure 3B). An analysis of the Venn diagram of differential metabolites indicated that TBSS vs. NC and SM vs. TBSS share 12 common differential metabolites (Figure 3C). Notable increases in metabolites such as Oleoylcarnitine, Docosapentaenoic acid, L-Tryptophan, Tetracosahexaenoic acid, 1-(Cyclohexylmethyl) proline, Sphinganine, Indoleacetaldehyde, and 4-Guanidinobutyric acid were observed in TBSS vs. NC platelets. Conversely, there was a significant reduction in metabolites including L-Kynurenine, Oleic acid, and 2-Arachidonylglycerol. Following SM administration, the levels of differential metabolites related to SM vs. TBSS returned toward normal levels (Table 1).

**Table 1:** Common differential metabolites between TBSS vs. NC and SM vs. TBSS groups.

No.	ID	MS2 name	Trend	
			TBSS vs. NC	SM vs. TBSS
1	98	Oleoylcarnitine	Up	Down
2	206	L-Kynurenine	Down	Up
3	410	Docosapentaenoic acid	Up	Down
4	432	L-Tryptophan	Up	Down
5	438	Tetracosahexaenoic acid	Up	Down
6	595	1-(Cyclohexylmethyl) proline	Up	Down
7	940	Sphinganine	Up	Down
8	982	Indoleacetaldehyde	Up	Down
9	1070	Oleic acid	Down	Up
10	1111	4-Guanidinobutyric acid	Up	Down
11	1181	2-Arachidonylglycerol	Down	Up
12	1241	Xanthosine	Up	Down

The differential metabolites KEGG enrichment pathways for the TBSS vs. NC and SM vs. TBSS groups are illustrated in Figures 3D,E. The DA Score reflects the overall changes in all metabolites within the metabolic pathway. A score of 1 indicates the upregulation of all annotated differential metabolites in the pathway, while a score of −1 indicates their downregulation. The length of the line segments represents the absolute value of the DA Score. The size of the dots corresponds to the number of differential metabolites annotated in the pathway, with larger dots indicating a greater number of differential metabolites. The dots are distributed to the right of the central axis; the longer the segment, the more pronounced the overall expression is. Conversely, on the left side of the central axis, longer segments indicate a greater tendency for the overall expression of the pathway to be downregulated.

The differential metabolite enrichment pathways in TBSS vs. NC were upregulated in Tryptophan metabolism, Arginine and Proline metabolism, D-Amino acid metabolism, Sphingolipid metabolism, and Purine metabolism, while they were downregulated in the biosynthesis of unsaturated fatty acids. In the comparison of SM vs. TBSS, the enriched pathways in Arginine and Proline metabolism, Arginine biosynthesis, beta-Alanine metabolism, Tryptophan metabolism, and Purine metabolism were downregulated. Additionally, pathways in Glycine, Serine, and Threonine metabolism, Cysteine metabolism, the Pentose phosphate pathway, Glyoxylate and Dicarboxylate metabolism, the biosynthesis of unsaturated fatty acids, Glutathione metabolism, and Glycerolipid metabolism were also downregulated.

### 3.6 Platelet proteomic analysis

#### 3.6.1 Differential protein screening and enrichment analysis

**Figure 4:**
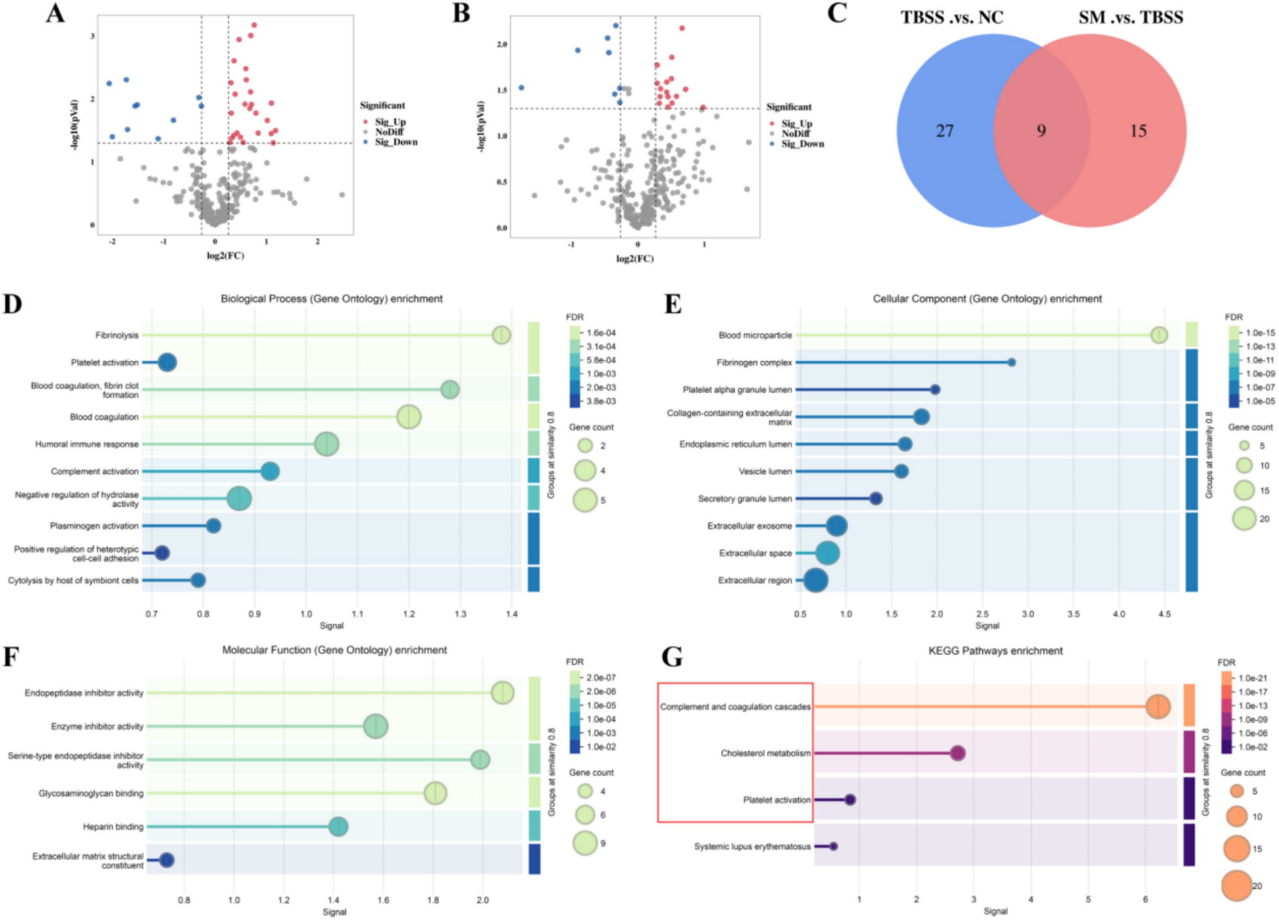
**(A,B)** Display volcano plots of TBSS vs. NC, SM vs. TBSS, respectively. In these plots, red indicates upregulated proteins, while green denotes downregulated proteins. **(C)** Venny of the differentially expressed proteins. Results of the differential proteins GO enrichment analysis **(D/E/F)** and the KEGG enrichment analysis **(G)**. In the bubble plot, the color of the circles represents the significance of enrichment, while the size of the circles reflects the number of differentially expressed proteins that are enriched.

To screen for differential protein expression among NC, TBSS, and SM, we collected mass spectrometry data from nine samples using the timsTOF_HT mass spectrometer in DIA mode, detecting a total of 4,663 peptides and 316 proteins. We applied screening criteria of FC > 1.2 or FC < 1/1.2 and *p* < 0.05, to identify significant upregulation and downregulation of differential proteins. TBSS vs. NC, we identified 36 differentially expressed proteins, of which 26 were upregulated and 10 were downregulated (Figure 4A). Details of the upregulated and downregulated proteins are presented in Supplementary Table S1. In the comparison of SM vs. TBSS, we identified 24 differentially expressed proteins, with 16 upregulated and 8 downregulated (Figure 4B). Details of these proteins are provided in Supplementary Table S2. A Venn diagram illustrating the differential protein expression between the groups (Figure 4C).

GO annotation and enrichment analysis were conducted for TBSS vs. NC and SM vs. TBSS. Figure 4D illustrates biological processes (BP) such as fibrinolysis, coagulation regulation, blood coagulation, fibrin clot formation, and plasminogen activation. Figure 4E presents cellular components (CC), including blood particles, fibrinogen complexes, platelet particles, collagen-containing cells, and the endoplasmic reticulum. Figure 4F details molecular functions (MF) such as endopeptidase inhibitor activity, enzyme inhibitor activity, serine-type endopeptidase inhibition, active glycosaminoglycan binding, heparin binding, and the structure of the extracellular matrix.

KEGG enrichment analysis was conducted to assess whether there is a significant enrichment trend among various functional categories based on the number of differentially expressed proteins. The results, illustrated in Figure 4G, indicate that the differentially expressed proteins exhibited significant differences in the complement and coagulation cascade, cholesterol metabolism, and platelet activation pathways.

#### 3.6.2 Interaction network analysis of differentially expressed proteins

**Figure 5:**
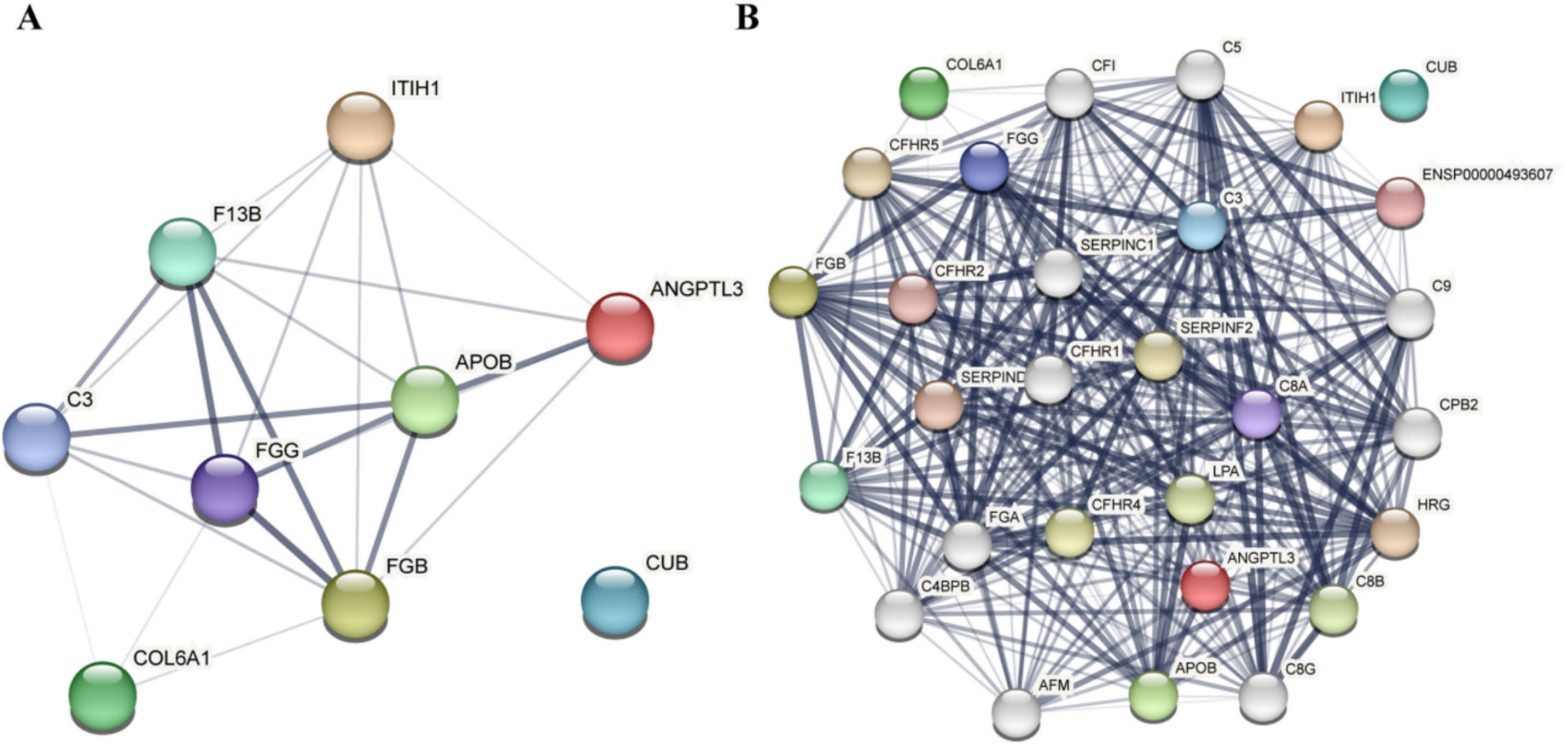
Results of the differential protein interaction network analysis. **(A)** Analysis results of the nine common differential proteins. **(B)** Analysis results of the nine common differential protein association proteins.

The STRING database is a resource that predicts functional correlations between proteins. In this study, common differential proteins were analyzed using the STRING database to provide a comprehensive overview of interprotein correlations. Nine common differential proteins were examined in the STRING database to illustrate all protein interaction relationships, including nodes with no hidden interactions (Figure 5A). The results indicated that among the selected common differential proteins, ITIH1, ANGPTL3, F13B, APOB, C3, FGG, FGB, and COL6A1 exhibited no interactions with CUB.

### 3.7 PRM testing and verification

Based on the results of the differential protein interaction network analysis obtained from STRING, the “+ More” option revealed additional associated proteins (Figure 5B). These proteins form clusters of interaction with common differential proteins, and the presence of 28 target proteins was confirmed through Parallel Reaction Monitoring (PRM).

**Figure 6:**
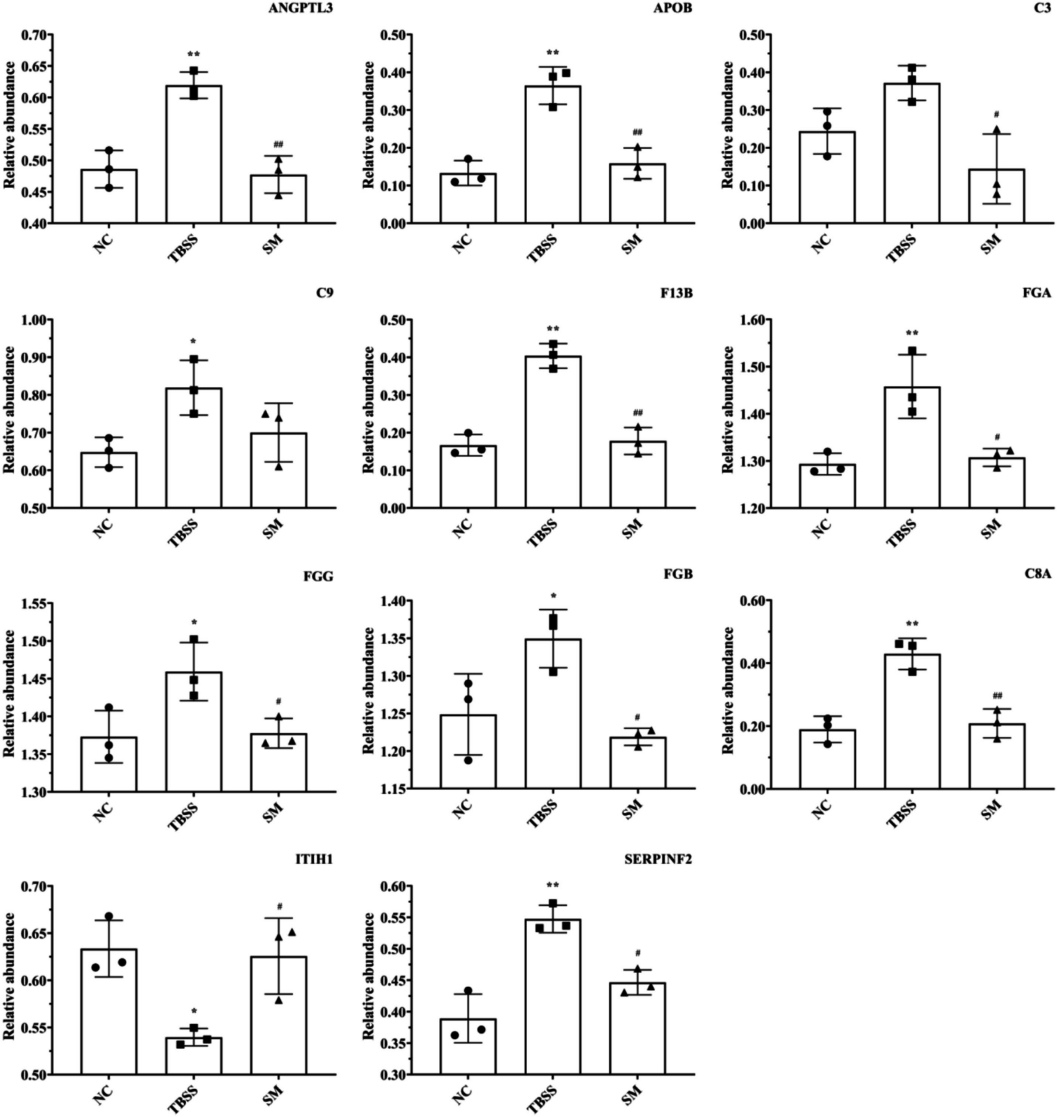
PRM analysis of protein determination results. “ITIH1” Inter-alpha-trypsin inhibitor heavy chain H1, “ANGPTL3” Angiopoietin-related protein 3, “APOB” Apolipoprotein B-100, “F13B” Coagulation factor XIII B chain, “FGB” Fibrinogen beta chain, “C3” Complement C3, “FGG” Fibrinogen gamma chain, “SERPINF2” Alpha-2-antiplasmin, “FGA” Fibrinogen alpha chain, “C9” Complement C9, “C8A” Complement C8 alpha chain.

PRM formal testing involved 21 protein peptides, which provided reliable identification information and demonstrated good chromatographic separation of 28 target proteins. Following formal testing with PRM mass spectrometry, the expression trends of seven proteins—ITIH1, ANGPTL3, APOB, F13B, FGB, C3, and FGG—were found to be consistent with the results from 4D-DIA proteomics in the NC, TBSM, and SM groups. Additionally, four associated proteins (SERPINF2, FGA, C9, and C8A) that interacted with common differential proteins exhibited the effects of TBSS after SM treatment, as illustrated in Figure 6. Compared to the NC group, the TBSS group showed significant increases in ANGPTL3, APOB, F13B, FGB, C3, FGG, SERPINF2, FGA, C9, and C8A (*p* < 0.01), while ITIH1 significantly decreased (*p* < 0.05). In contrast, in the SM group, ANGPTL3, APOB, F13B, FGB, C3, FGG, SERPINF2, FGA, C9, and C8A exhibited extremely significant decreases (*p* < 0.01), whereas ITIH1 significantly increased (*p* < 0.05).

## 4 Discussion

According to traditional Chinese medicine, injuries from falls and torn tendons can lead to internal damage to the meridians and disrupt blood flow. This disruption causes blood to stagnate within the meridians, a condition that the treatment principles aim to address by promoting blood circulation, unblocking the meridians, and alleviating swelling and pain (2). Following trauma, the body generates a series of excessive stress responses that disrupt the delicate balance between coagulation and fibrinolysis. Research has shown that TCM can reduce blood stasis by inhibiting platelet activation and fibrinolysis in rats (14). The parameters TT, PT, APTT, and FIB are critical indicators of the efficiency of the plasma coagulation pathway (15–17). Furthermore, the administration of SM improved the abnormal coagulation function induced by mold formation, suggesting that SM influences both the intrinsic and extrinsic coagulation pathways and promotes fibrin formation, which aligns with existing literature (18–21). Disruption of the dynamic balance between t-PA and PAI-1 results in a decreased local rate of fibrin breakdown within the vessel, leading to fibrin deposition and the formation of congestion (22). In this study, SM may alleviate blood stasis caused by TBSS by enhancing the activity of the fibrinolytic system. The literature indicates that certain active ingredients in TCM exhibit significant anti-blood stasis effects in patients through mechanisms such as anticoagulation, antiplatelet activation, and antifibrinolytic effects, which involve the modulation of t-PA and PAI-1 levels in animal models (23–25).

Platelets play a crucial role in BSS, and the mass accumulation of platelets can lead to pathological congestion (26, 27). The levels of TXB_2_, 6-keto-PGF1α, and TXB_2_/6-keto-PGF1α are three important factors in assessing the antiplatelet effect. Previous studies have verified that TCM maintains the balance of TXA_2_ and PGI_2_ in acute blood stasis model rats (28, 29). This research indicates that SM regulates the levels of TXB_2_ and 6-keto-PGF1α to reduce platelet aggregation and coagulation. FN influences the adhesion of platelets to fibrin (30), while PF4 promotes platelet aggregation (31). Additionally, *β*-TG inhibits prostacyclin synthase, leading to increased platelet aggregation, and β-TG level serve as an indicator of platelet activity (32). These findings suggest that SM administration inhibits platelet aggregation associated with TBSS, reduces the extent of platelet activation, and alleviates the severity of blood stasis. Furthermore, some studies have shown that SalB and Tanshinone IIA (Tan IIA), the active components of SM, also inhibit platelet function and aggregation caused by various pathogenic factors (9, 33).

Our study focused on the alterations in endogenous metabolites and differential proteins within platelets, elucidated how SM inhibited platelet aggregation and activation. The results indicated that 12 metabolites were significantly altered following SM administration, primarily enriched in pathways related to amino acid metabolism, arginine and proline metabolism, sphingolipid metabolism, purine metabolism, and unsaturated fatty acid metabolism. Additionally, seven differential proteins returned to near-normal levels after SM administration, with these proteins being associated with the complement and coagulation cascade, cholesterol metabolism, and platelet activation processes.

Tryptophan, a significant metabolite, plays a crucial role in several physiological processes within the body (34). It serves as the synthetic precursor of serotonin (5-HT) (35, 36). However, elevated levels of 5-HT in the blood can lead to platelet aggregation, resulting in a hypercoagulable state (37). The results indicate that the regulation of tryptophan metabolism may be a key mechanism through which SM promotes blood circulation and alleviates blood stasis. Additionally, xanthine catalyzes the production of uric acid, and elevated levels of uric acid are associated with endothelial dysfunction, increased platelet adhesion, altered blood rheological properties, and enhanced platelet aggregation (38). The increase in xanthine levels in platelets may further contribute to elevated uric acid levels, potentially leading to metabolic disorders. However, xanthine levels returned to normal following the administration of SM. Fatty acid metabolism is a crucial metabolic process that maintains the body’s energy homeostasis. Both docosapentaenoic acid and 24-sahhexaenoic acid are *ω*-3 fatty acids that inhibit the conversion of arachidonic acid to thromboxane, thereby reducing platelet aggregation (39). The abnormal alterations in fatty acid components in platelets observed in the TBSS model group disrupted normal platelet function. However, following SM administration, these components were normalized, suggesting that the regulation of fatty acid metabolism may be one of the mechanisms through which SM promotes blood circulation and alleviates blood stasis.

The complement and coagulation cascades can activate one another (40), providing an appropriate innate response to injury, thereby limiting congestion and infection while promoting healing (41). In this study, F13B, FGB, C3, FGG, FGA, C9, C8A, and SERPINF2 were significantly enriched in the coagulation pathway. Previous research has demonstrated that the binding of activated platelets to C3b and C1q triggers the bypass pathway, activating the complement system (42–44). According to 4D-DIA and PRM analyses, SM facilitates blood circulation and alleviates blood stasis by regulating the complement and coagulation cascades. The TBSS model exhibited slow blood flow and increased levels of FGA, FGB, and FGG, which encode FIB. These increases are associated with higher concentrations of FIB and enhanced hydrolysis due to elevated thrombin levels, thereby exacerbating coagulation (45). Following the administration of SM, levels of FGA, FGB, and FGG decreased, indicating that SM mitigated the coagulation process by regulating FIB concentration and inhibiting both platelet activation and aggregation.

The present study utilized metabolomics based on the LC/Q-TOF-MS platform and 4D-DIA proteomic analyses to elucidate the intervention mechanisms of SM on TBSS. However, this study has several limitations. While key proteins identified through proteomic analysis were quantitatively detected using PRM, the identified potential metabolite markers have not undergone further validation. Therefore, to more effectively elucidate the therapeutic mechanisms of SM in promoting blood circulation and alleviating blood stasis, our current findings should be validated through additional research aimed at identifying potential metabolite markers, related regulatory genes, and key signaling pathways at the molecular and cellular levels.

## 5 Conclusion

In this study, the effects of SM were evaluated by establishing a cat model of TBSS. SM regulates swelling and pain, inflammatory responses, abnormalities in the coagulation and fibrinolytic systems, as well as platelet aggregation and activation. Through metabolomic and proteomic analyses, it was found that SM inhibited the aggregation and activation processes of TBSS platelets by modulating various physiological processes, including tryptophan metabolism, purine metabolism, fatty acid metabolism, the complement and coagulation cascades, and platelet activation.

## Data Availability

The original contributions presented in the study are publicly available. This data can be found at: https://ngdc.cncb.ac.cn/omix; OMIX009811.
