# Supplementary material for: Integrative metabolomics and proteomics reveal the effects and mechanisms of Salvia miltiorrhiza in alleviating traumatic blood stasis syndrome
Source: Front Vet Sci. 2025 Apr 25;12:1579790. doi: 10.3389/fvets.2025.1579790 (PMC12083185; doi:10.3389/fvets.2025.1579790)
Supplement: Supplementary file 1 [file Data_Sheet_1.docx]

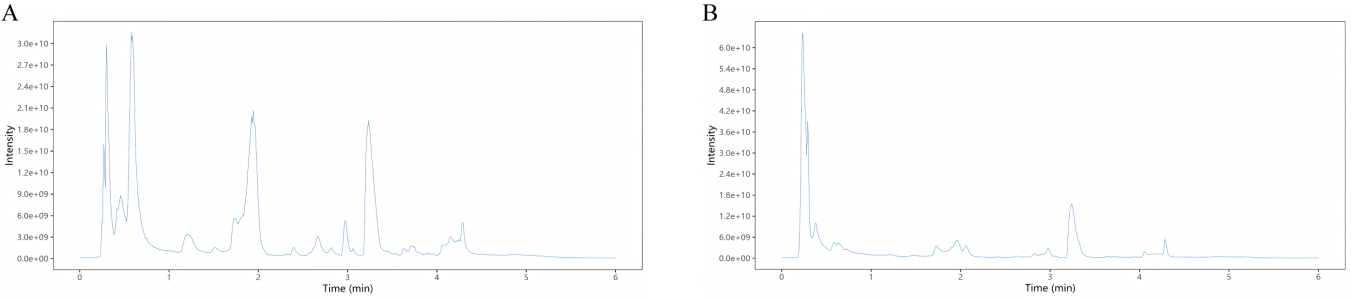


Fig.S1 TIC diagram of UHPLC-OE-MS for positive (A) and negative (B) mode


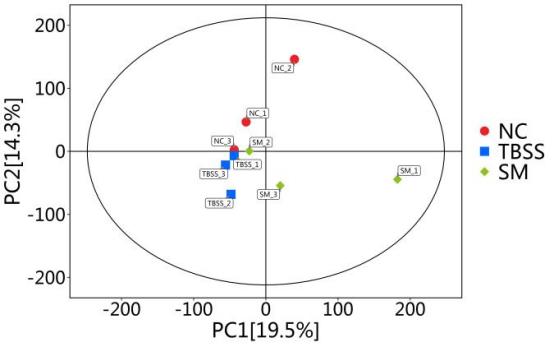


Fig.S2 Scatter plot of the PCA model between the NC *vs*. TBSS *vs*. SM groups. Each scatter point represents a sample, with the color and shape indicating different groups. When the distribution of sample points is closer together, it suggests that the types and contents of metabolites in those samples are more similar. Conversely, when the sample points are farther apart, it indicates a greater difference in the overall metabolic levels.


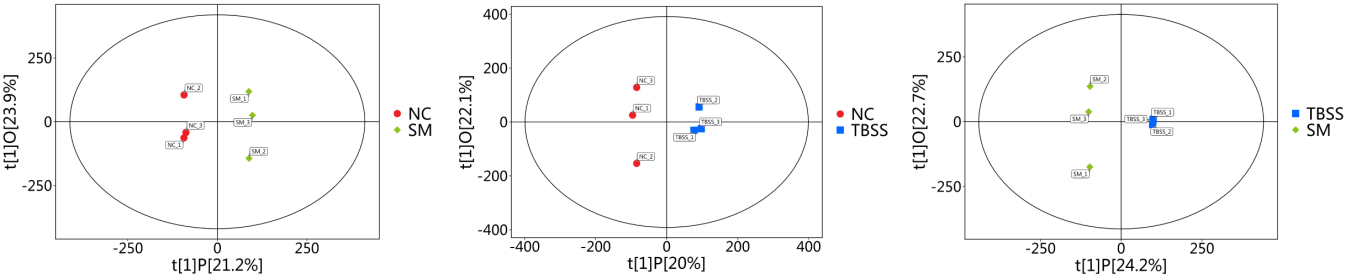


Fig.S3 OPLS-DA graph. In the abscissa, t[1]P represents the first principal component score, which illustrates the differences between sample groups. Longitudinal t[1]O represents the orthogonal principal component score, highlighting the differences within the sample group. Each scatter point represents a sample, with the shape and color of the scatter indicating different test groups. The transverse distance between the samples reflects the magnitude of the differences, while the longitudinal distance indicates the repeatability within the group.

TableS1 Differential proteins statistical results of TBSS vs. NC

| **NO.** | **Protein name** | **ID** | **Gene Name** | **FC** | ***p*** | **Trend** |
| --- | --- | --- | --- | --- | --- | --- |
| 1 | Coagulation factor XIII A chain | A0A2I2U5J7_FELCA | F13A1 | 1.6982 | 0.0007 | Up |
| 2 | Transferrin receptor protein 1 | TFR1_FELCA | TFRC | 1.6197 | 0.0010 | Up |
| 3 | Alpha-2-HS-glycoprotein | A0A337SD37_FELCA | AHSG | 1.3846 | 0.0011 | Up |
| 4 | Ig-like domain-containing protein | A0A2I2U5M6_FELCA | Ig-like5M6 | 1.2935 | 0.0025 | Up |
| 5 | Vascular cell adhesion molecule 1 | M3WED4_FELCA | VCAM1 | 1.5149 | 0.0033 | Up |
| 6 | Lumican | A0A2I2U619_FELCA | LUM | 1.5245 | 0.0050 | Up |
| 7 | CUB domain-containing protein | A0A5F5Y006_FELCA | CUB | 1.2411 | 0.0055 | Up |
| 8 | Collagen type VI alpha 3 chain | A0A337RW59_FELCA | COL6A3 | 1.6159 | 0.0077 | Up |
| 9 | Intercellular adhesion molecule 1 | M3WBF1_FELCA | ICAM1 | 1.3126 | 0.0084 | Up |
| 10 | Ig-like domain-containing protein | M3WX66_FELCA | Ig-likeX66 | 2.1396 | 0.0116 | Up |
| 11 | Angiopoietin like 3 | M3WET8_FELCA | ANGPTL3 | 1.4978 | 0.0120 | Up |
| 12 | Complement component 4 binding protein alpha | A0A5F5Y5F1_FELCA | C4BPA | 1.6362 | 0.0122 | Up |
| 13 | Ig-like domain-containing protein | A0A2I2U1K0_FELCA | Ig-like1K0 | 1.5976 | 0.0141 | Up |
| 14 | Ig-like domain-containing protein | A0A5F5XCM6_FELCA | Ig-likeCM6 | 1.7387 | 0.0167 | Up |
| 15 | Fibulin-1 | A0A337SRF9_FELCA | FBLN1 | 1.2458 | 0.0168 | Up |
| 16 | Creatine kinase B-type | A0A337S4U0_FELCA | CKB | 2.0240 | 0.0219 | Up |
| 17 | Apolipoprotein B | A0A337SHX7_FELCA | APOB | 2.2658 | 0.0317 | Up |
| 18 | Serpin family F member 1 | M3W5N1_FELCA | SERPINF1 | 1.3496 | 0.0345 | Up |
| 19 | Joining chain of multimeric IgA and IgM | A0A5F5XC52_FELCA | JCHAIN | 1.7912 | 0.0347 | Up |
| 20 | Lipocalin/cytosolic fatty-acid binding domain-containing protein | A0A5F5XI37_FELCA | LOC109496317 | 2.1342 | 0.0353 | Up |
| 21 | Coagulation factor XIII B chain | M3W3B5_FELCA | F13B | 1.2901 | 0.0377 | Up |
| 22 | Haptoglobin | A0A5F5XGD0_FELCA | HP | 1.4097 | 0.0404 | Up |
| 23 | Fibrinogen beta chain | M3WII3_FELCA | FGB | 1.2579 | 0.0414 | Up |
| 24 | Complement C3 | A0A5F5Y2U5_FELCA | C3 | 1.4624 | 0.0490 | Up |
| 25 | Fibrinogen gamma chain | A0A5F5XGZ0_FELCA | FGG | 1.2205 | 0.0491 | Up |
| 26 | Collagen type VI alpha 1 chain | M3WLD9_FELCA | COL6A1 | 2.1931 | 0.0497 | Up |
| 27 | Keratin 74 | M3VUF9_FELCA | KRT74 | 0.3019 | 0.0049 | Down |
| 28 | Keratin, type I cytoskeletal 17 | A0A5F5XLA0_FELCA | KRT17 | 0.2397 | 0.0057 | Down |
| 29 | Inter-alpha-trypsin inhibitor heavy chain H1 | M3WN59_FELCA | ITIH1 | 0.8044 | 0.0095 | Down |
| 30 | Keratin 80 | M3X1L9_FELCA | KRT80 | 0.3502 | 0.0123 | Down |
| 31 | Keratin, type I cytoskeletal 10 | M3VXR1_FELCA | KRT10 | 0.3400 | 0.0130 | Down |
| 32 | Coagulation factor XI | M3VWV2_FELCA | F11 | 0.8319 | 0.0130 | Down |
| 33 | Ras association domain family member 2 | A0A5F5XQ13_FELCA | RASSF2 | 0.5708 | 0.0217 | Down |
| 34 | Keratin 3 | M3VUG8_FELCA | KRT3 | 0.3068 | 0.0305 | Down |
| 35 | Keratin 17 | A0A5F5Y768_FELCA | KRT17 | 0.2501 | 0.0397 | Down |
| 36 | Keratin 9 | A0A5F5XQZ2_FELCA | KRT9 | 0.4638 | 0.0426 | Down |

TableS2 Differential proteins statistical results of SM vs. TBSS

| **NO.** | **Protein name** | **ID** | **Gene Name** | **FC** | ***p*** | **Trend** |
| --- | --- | --- | --- | --- | --- | --- |
| 1 | Inter-alpha-trypsin inhibitor heavy chain H1 | M3WN59_FELCA | ITIH1 | 1.2222 | 0.0265 | Up |
| 2 | Glyceraldehyde-3-phosphate dehydrogenase | G3P_FELCA | GAPDH | 1.4221 | 0.0139 | Up |
| 3 | Galectin-3-binding protein | A0A2I2U679_FELCA | CANT1 | 1.3479 | 0.0333 | Up |
| 4 | Coagulation factor V | M3W922_FELCA | F5 | 1.2661 | 0.0304 | Up |
| 5 | Protein S100 | M3VWD6_FELCA | S100A8 | 1.9710 | 0.0486 | Up |
| 6 | Apolipoprotein A-I | A0A337S837_FELCA | APOA1 | 1.5831 | 0.0067 | Up |
| 7 | Transferrin | A0A5F5Y527_FELCA | TF | 1.2490 | 0.0440 | Up |
| 8 | Complement C1q subcomponent subunit A | M3WS63_FELCA | C1QA | 1.2588 | 0.0372 | Up |
| 9 | Ig-like domain-containing protein | A0A337SA21_FELCA |  | 1.6427 | 0.0309 | Up |
| 10 | Transferrin | M3WBQ5_FELCA | TF | 1.4259 | 0.0437 | Up |
| 11 | Albumin | A0A2I2U7Y0_FELCA | ALB | 1.4164 | 0.0237 | Up |
| 12 | Carbonic anhydrase | A0A5F5XMJ0_FELCA | CA2 | 1.3678 | 0.0374 | Up |
| 13 | Transferrin | A0A5F5XNI1_FELCA | TF | 1.3494 | 0.0257 | Up |
| 14 | Glycoprotein V platelet | A0A2I2U027_FELCA | GP5 | 1.4935 | 0.0369 | Up |
| 15 | CUB domain-containing protein | A0A5F5Y006_FELCA | CUB | 1.2228 | 0.0168 | Up |
| 16 | Collagen type VI alpha 1 chain | M3WLD9_FELCA | COL6A1 | 1.3661 | 0.0484 | Up |
| 17 | Gelsolin | M3VZP6_FELCA | GSN | 0.8280 | 0.0431 | Down |
| 18 | Vanin 1 | M3WEJ3_FELCA | VNN1 | 0.2974 | 0.0297 | Down |
| 19 | Angiopoietin like 3 | M3WET8_FELCA | ANGPTL3 | 0.7290 | 0.0085 | Down |
| 20 | Apolipoprotein B | A0A337SHX7_FELCA | APOB | 0.5348 | 0.0116 | Down |
| 21 | Coagulation factor XIII B chain | M3W3B5_FELCA | F13B | 0.7853 | 0.0349 | Down |
| 22 | Fibrinogen beta chain | M3WII3_FELCA | FGB | 0.7385 | 0.0123 | Down |
| 23 | Complement C3 | A0A5F5Y2U5_FELCA | C3 | 0.7943 | 0.0063 | Down |
| 24 | Fibrinogen gamma chain | A0A5F5XGZ0_FELCA | FGG | 0.8267 | 0.0300 | Down |
